# The Berkeley Dry Eye Flow Chart: A fast, functional screening instrument for contact lens-induced dryness

**DOI:** 10.1371/journal.pone.0190752

**Published:** 2018-01-24

**Authors:** Andrew D. Graham, Erika L. Lundgrin, Meng C. Lin

**Affiliations:** Clinical Research Center, School of Optometry, University of California, Berkeley, California, United States of America; University of Alabama at Birmingham, UNITED STATES

## Abstract

**Purpose:**

In this article, we introduce a novel flow chart-based screening tool for the categorization of contact lens-induced dryness (CLIDE) and its impact on daily visual activities: the Berkeley Dry Eye Flow Chart (DEFC).

**Methods:**

One hundred thirty (130) experienced soft contact lens wearers discontinued lens wear for 24 hrs, passed a baseline screening and eye health examination, completed the Ocular Surface Disease Index (OSDI) then were dispensed fresh pairs of their habitual lenses. After 6 hrs of wear, subjects were administered a battery of symptom questionnaires, and underwent non–invasive tear breakup time (NITBUT) measurement, grading of distortion in reflected topographer mires, grading of lens surface wettability, and a fluorescein examination of the ocular surface. Subjects returned after at least 48 hrs and repeated all assessments after 6 hrs of wear of a second fresh pair of habitual lenses.

**Results:**

The repeatability of the DEFC between visits was within 1%, and Limits of Agreement and Coefficient of Repeatability were comparable to those of the other CLIDE assessments. Higher DEFC score was significantly related to shorter pre-lens NITBUT, higher OSDI score, and higher Visual Analog Scale (VAS) ratings of average and end-of-day severity and frequency of dryness (all p < 0.001). For CLIDE as diagnosed based on DEFC score, the highest sensitivities and specificities were achieved by the OSDI and VAS ratings; pre-lens NITBUT exhibited good sensitivity but poor specificity. The optimum pre-lens NITBUT diagnostic threshold was found to be ≤ 2.0 sec for debilitating CLIDE, and the OSDI threshold was ≥ 11.4.

**Conclusions:**

The DEFC provides a means of quickly categorizing CLIDE patients based on severity and frequency of symptoms, and on the degree to which symptoms impact daily life. The DEFC has several potential advantages as a CLIDE screening and monitoring tool, has good repeatability, and is significantly related to commonly employed clinical assessments for CLIDE.

## Introduction

Contact-lens-induced dryness (CLIDE) is a condition with a multifaceted etiology and widely varying presentation, with no consensus among researchers or clinicians as to how it should be diagnosed [1–4]. The International Workshop on Contact Lens Discomfort (CLD) classified dryness during contact lens wear as one form of CLD, reserving the term “contact lens-related dry eye” for a pre-existing dry eye (DE) condition which (rather confusingly) “may or may not be exaggerated” during lens wear [2]. Most studies to date have not attempted to rigorously define CLIDE but rather have simply examined dryness symptoms that occur during lens wear or have compared “symptomatic” and “asymptomatic” groups of subjects defined in various ways [5–10]. Less commonly, CLIDE has been described as the presence of dryness symptoms only during lens wear, or that resolve when lenses are removed [11–14].

CLIDE symptoms have typically been assessed using questionnaire instruments developed for DE and not specifically designed or validated for contact lens wear. These include the Ocular Surface Disease Index (OSDI) [15,16], the Dry Eye Questionnaire (DEQ) [13], the Standard Patient Evaluation of Eye Dryness (SPEED) [17], the Impact of Dry Eye on Everyday Life (IDEEL) questionnaire [18], the Subjective Evaluation of Symptoms of Dryness (SESoD) [19], and McMonnies’ Dry Eye Index (DEI) [20–22], The OSDI has been extensively validated for DE patients, and although not designed or specifically validated for CLIDE [4], has been widely used in its study. McMonnies DEI also was designed and validated for DE patients, but was later validated in a study of 70 contact lens wearers [6].

Of the few published questionnaire instruments that were developed specifically for CLIDE (as distinct from DE in general), the 8-item short form of the Contact Lens Dry Eye Questionnaire (CLDEQ-8) is the most widely employed [13,23,24]. The CLDEQ-8 includes 5-point ordinal rating scale items for the frequency and end-of-wearing-period intensity of discomfort, dryness, and changeable, blurry vision during lens wear, as well as for frequency of desire to close the eyes and of contact lens removal due to symptoms, all of which are then summed for a total CLIDE score. A subset of CLDEQ-8 questions regarding the frequency and end-of-wearing-period intensity of symptoms has been employed by Young [25,26] to assign a categorization of CLIDE-positive, CLIDE-negative or marginal.

**Fig 1:**
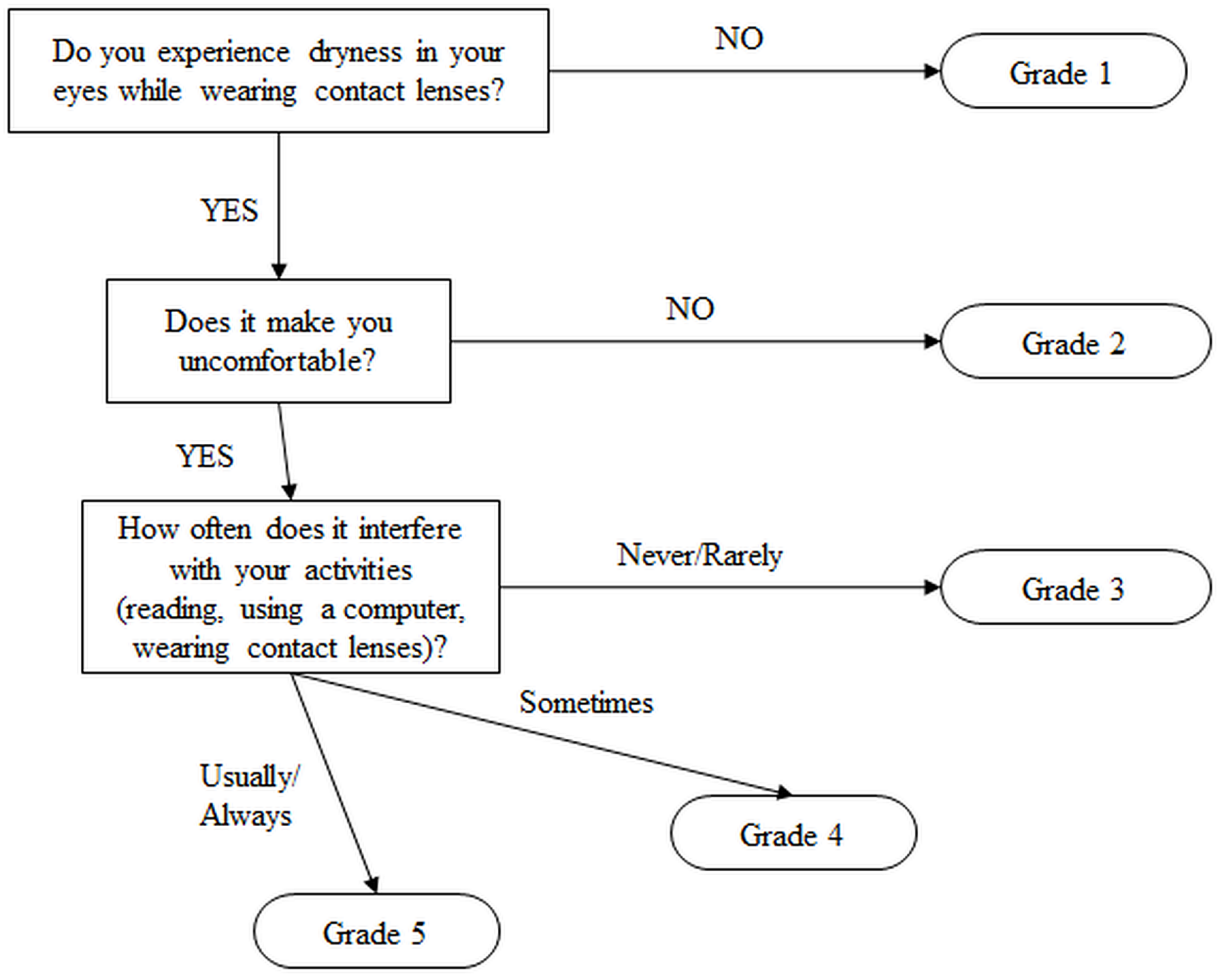
The Berkeley Dry Eye Flow Chart (DEFC).

The purpose of this study is to describe a new flow chart-based questionnaire for the categorization of CLIDE: the Berkeley Dry Eye Flow Chart (DEFC; Fig 1) [27,28]. The DEFC categorizes individuals into one of five CLIDE types based on the presence/absence of dryness sensation during lens wear over the previous week, the perception of discomfort caused by dryness symptoms when present, and the frequency with which dryness symptoms interfere with daily activities such as reading or using the computer (interference may include having to interrupt the activity to use eye drops, having to remove contact lenses, or having to stop the activity altogether). The DEFC was developed by U.C. Berkeley Clinical Research Center staff, including optometrists, clinical researchers, and biostatisticians, through discussions with colleagues in clinical practice who felt that numerical grading of symptoms or Likert-style checkbox questionnaires were insufficient to capture how a symptomatic patient really felt. While an internal mental state is a latent trait, it may be made manifest (and measured) in a change of expression or behavior related to the symptoms. From a clinical perspective, the extent to which symptoms interfere with daily activities is a good indicator of the severity of the problem, and suggestive of how it should be further treated. From a research perspective, it was determined that the instrument should be short and easy to complete, thus reducing the potential for misclassification bias. It was finally determined that the most promising design would be a flow-chart questionnaire, with binary items related to the presence or absence of symptoms, their severity and frequency, and their impact on daily life.

There are several potential advantages inherent in the design of the DEFC. It is very simple to read and understand, reducing the potential for later misdiagnosis due to incorrectly completed questionnaires from patients who are fatigued or confused by a longer or more difficult instrument, and reducing the potential for bias in clinical studies due to over-selecting for the capabilities required to successfully complete longer, more difficult questionnaires [29]. Because the DEFC score incorporates aspects of symptom presence, sensation, severity, frequency and especially impact on everyday activities, different types of functional CLIDE can be identified by setting the DEFC score at appropriate diagnostic thresholds. For example, a researcher may wish to assign potential clinical trial subjects to either a control group with no CLIDE symptoms (DEFC score < 2) or a CLIDE group with symptoms spanning the entire range encountered in the target population (DEFC score ≥ 2); a clinician may wish to screen patients (n.b., with further clinical assessment before initiating actual treatment) whose symptoms require no or only mild intervention such as occasional application of artificial tears (DEFC score < 4) from those whose symptoms are severe enough to be debilitating and require more thorough investigation and possibly more aggressive treatment (DEFC score ≥ 4). Finally, the DEFC score provides a fast and easy way to gather a basic, functional, actionable reading of CLIDE status, which can be logistically important in facilitating large numbers of potential subjects being quickly pre-qualified in a clinical trial setting or classified for epidemiologic study, or large numbers of patients being efficiently pre-screened in an institutional setting (e.g., an on-site screening at a senior care facility) [30,31].

Our goals in this paper are: (1) to establish the repeatability of the the DEFC and to compare its performance with that of other known subjective and clinical measures of CLIDE; (2) to determine whether several clinical tests and subjective symptom measures of CLIDE are related to the DEFC score, and to determine their effect sizes and significance of association; (3) to determine the diagnostic performance (sensitivity and specificity, optimum threshold) of these subjective and clinical measures for a DEFC-based diagnosis of CLIDE. We will present this analysis for the two different examples of DEFC-based CLIDE diagnosis described above: No Symptoms vs. Any Symptoms, and No/Mild Symptoms vs. Debilitating Symptoms. These findings will serve to illuminate whether and to what degree clinical outcomes and subjective symptom measures are reflected in different types of CLIDE, based on the functional classification offered by the DEFC. With its characteristics and performance documented, the DEFC could prove to be a useful addition to the investigational and screening tools available for CLIDE, and to its diagnosis and management in the clinical setting.

## Materials and methods

### Subjects

Subjects were recruited from the University of California, Berkeley campus and surrounding community through posted flyers and direct referrals. Potential subjects were soft contact lens wearers 18 years of age or older who had had eye examinations within the previous 2 years. Eligible subjects were free of any ocular surface pathology or health conditions with ocular manifestations, and were not taking any prescription or over-the-counter medications that could affect the ocular surface or tear film.

### Study protocol and procedures

Subjects who passed an initial eligibility screening by telephone and elected to participate were instructed to report to the U.C. Berkeley Clinical Research Center (CRC) for a baseline examination and final eligibility determination, wearing their habitual lenses. Upon arrival at the baseline visit, subjects completed a background questionnaire to collect demographic data as well as medical and contact lens histories, and to complete the Ocular Surface Disease Index (OSDI). Subjects then completed the DEFC followed by 100-point rating scale questionnaires on the severity and frequency of all-day and end-of-day dryness symptoms, presented as Visual Analog Scales (VAS) [32,33]. For dryness severity, a rating of 0 = No Dryness and a rating of 100 = Severe Dryness; for dryness frequency, a rating of 0 = Never and a rating of 100 = All the Time. VAS ratings were given by the subject making a vertical mark through a 10 cm horizontal line with only the end points labelled as described, to indicate where his or her eyes fell on the continuum of symptoms, the location of which was automatically determined by scanning software (Teleform, Hewlett Packard, Palo Alto, CA, USA).

Eligible subjects were then scheduled for 2 follow-up visits, at least 48 hrs apart. Subjects were dispensed 2 fresh pairs of their habitual lenses, and were instructed to insert a fresh pair of lenses at least 6 hrs prior to each follow-up visit. At these visits, subjects first completed all dryness symptom questionnaire instruments, following which, a corneal topographer (E300, Medmont International Pty. Ltd., Melbourne, Victoria, AUS) was used to measure non-invasive tear breakup time (NITBUT). Breakup time was recorded at the first sign of a break or distortion in the reflected mires. Three measurements per eye were taken and averaged, with at least 30 seconds between measurements to allow blinking to refresh the tear film. Following the NITBUT measurement, in a mild form of “stress test”, subjects were instructed to blink and then hold the eyes open, and at 10 sec the severity of distortion or haze in the reflected topographer mires was graded on a 0–4 scale (0 = No haze or distortion; 4 = Extreme haze or distortion) in the central, nasal, temporal and inferior zones. A slit lamp (SL 120, Carl Zeiss Meditec AG, Oberkochen, Baden-Württemberg, GER) with white light was then used to grade *in vivo* contact lens surface wettability on a 0–4 scale (0 = Non-wetting; 4 = Complete wetting). Following removal of the lenses and instillation of 2 μl of 0.35% sodium fluorescein to the bulbar conjunctiva, cobalt blue light and a Wratten #12 yellow barrier filter were employed to grade corneal and conjunctival staining according to the Brien Holden Vision Institute grading scales [34]. Conjunctival staining was graded separately in the nasal, temporal, superior and inferior quadrants. Corneal staining was graded separately in the 4 quadrants and in the central 5mm zone, in terms of the type, extent and depth of staining.

This study adhered to the tenets of the Declaration of Helsinki, was approved by institutional review board (U.C. Berkeley Committee for the Protection of Human Subjects) and was conducted in compliance with Health Insurance Portability and Accountability Act (HIPAA) guidelines for data safety and subject anonymity. All subjects provided written informed consent after a thorough description of the procedures, potential benefits, and risks involved in the study.

### Statistical methods

Repeatability of the DEFC instrument was assessed by comparing scores at the 2 visits, taken under as close to identical conditions as possible. The 2 visits were approximately 48 hours apart, both after at least 24 hours discontinuation of lens wear, insertion of a fresh pair of lenses, followed by 6 hours of wear. We examined the distribution of the differences (Visit 2 –Visit 1) in DEFC score, the mean difference, Limits of Agreement (LoA) [35], Difference vs. Means (DVM) plots [35], the % of scores within 1 point on the 5-point DEFC scale from visit to visit, and conducted a variance component analysis to estimate the within-subject variability in DEFC scores from which we calculated the Coefficient of Repeatability (CR) [36]. Strictly speaking, the true repeatability of an instrument requires measuring the identical phenomenon on multiple occasions under identical conditions with the same observer, which is not possible for subjective symptoms which can vary from day to day for some subjects, even under nearly identical conditions. We therefore conducted the same repeatability analysis on our other CLIDE measures (VAS ratings of symptoms, NITBUT, etc.) and converted those results to DEFC-equivalent scales, in order to show that the repeatability of the DEFC instrument, in the presence of inherently labile symptoms, is comparable to that of other commonly employed CLIDE assessments.

After repeatability assessment and a thorough exploratory analysis, a multivariable linear mixed effects modeling approach was taken to determine which clinical, laboratory and subjective measures were significantly related to DEFC score, accounting for potential within-subject correlations engendered by the repeated measures design. Because DEFC score is a subject-level variable, for eye-level variables such as tear breakup time or lens surface wettability, we examined grades for the worse eye (e.g., faster breakup time, poorer wettability), the better eye, the average of the two eyes and the sum of the two eyes as potential explanatory variables for DEFC score. For corneal and conjunctival staining, we also examined the grades zone-by-zone and summed over all zones.

After identifying which variables were significantly related to DEFC score, we employed Receiver Operating Characteristic (ROC) analysis [37] to estimate the sensitivities and specificities of these diagnostics to a DEFC-based determination of CLIDE status, for the two different thresholds (DEFC score ≥2 and ≥4) described above. In the ROC analysis we calculated the sensitivity and specificity using every possible value of each clinical and subjective measure as a diagnostic threshold, within the ranges we observed. We developed an algorithm to implement our adaptation of the method proposed by Emir, et al. [38] for estimating sensitivity and specificity in the presence of repeated measures. The optimum threshold was considered to be the value that resulted in sensitivity and specificity at the smallest Euclidean distance to the (0,1) point on the ROC plot (i.e., the point geometrically closest to 100% sensitivity and 100% specificity).

## Results

### Subject characteristics

One hundred thirty (130) subjects completed the study. Subjects ranged in age from 18 to 61 years, with a mean (SD) of 26.6 (9.7) years. A majority of subjects (n = 89, 68%) were female. Sixty Asian subjects (46%) and 70 non-Asian subjects (54%) were analyzed. The Asian group consisted of subjects of Chinese, Japanese, Korean, Southeast Asian or Pacific Islander descent. Non-Asian subjects included Caucasians, African-Americans, and Latinos. Subjects were all experienced soft contact lens wearers (38% silicone hydrogel, 62% hydrogel), with mean (SD) wearing times of 5.7 (1.7) days/week and 11.8 (3.4) hours/day. Further detail on the demographic makeup and baseline characteristics of our subjects is provided in Table 1.

**Table 1:** Baseline subject characteristics.

	Asian (n = 60)		Non-Asian (n = 70)	
	Female (n = 44)	Male (n = 16)	Female (n = 45)	Male (n = 25)
**Age (yrs)**	23.6 (6.9)	22.9 (5.4)	29.9 (11.6)	28.4 (10.2)
**Students**	34 (77.3%)	11 (68.8%)	23 (51.1%)	19 (76.0%)
**Computer (hrs/day)**	5.5 (3.7)	4.7 (2.6)	5.0 (2.6)	5.5 (3.0)
**CL Wear Hx (yrs)**	5.8 (6.5)	6.9 (12.5)	6.9 (7.6)	4.0 (4.8)
**CL Wear (hrs/day)**	11.6 (3.0)	12.3 (3.4)	12.1 (3.3)	11.4 (4.3)
**CL Wear (days/wk)**	5.9 (1.4)	5.1 (2.0)	5.6 (2.0)	5.5 (1.6)
**Eye Drops Use**	33 (75.0%)	8 (50.0%)	27 (60.0%)	16 (64.0%)

The n = 130 subjects are shown stratified on gender and on Asian vs. non-Asian descent. The mean (SD) in each stratum is shown for subject age, computer use, and contact lens (CL) wearing history and daily average wearing pattern. The number (n) and % of subjects in each stratum who are students and who use eye drops are also shown.

### Repeatability of the DEFC

**Fig 2:**
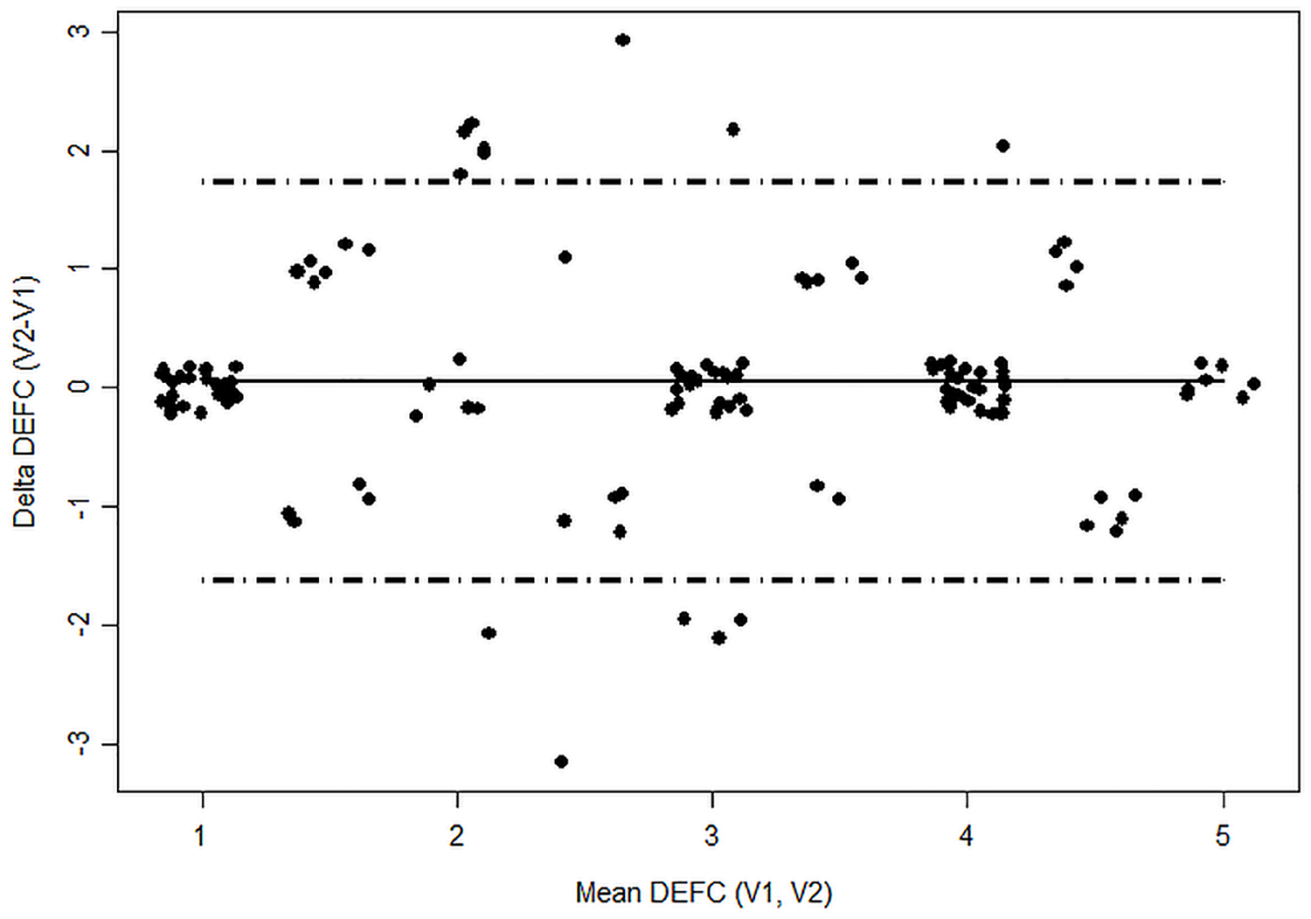
Difference-vs.-mean plot of DEFC scores. The mean difference in DEFC scores (Visit 2 –Visit 1) was 0.05 units (1.00%) on the 5-point DEFC scale. There was no pattern of dependence of the inter-visit difference on the size of the mean. The discrete ordinal DEFC scores are jittered to reveal coincident data.

The mean difference in DEFC scores (Visit 2 –Visit 1) was 0.05 units (1.00%) on the 5-point DEFC scale, and was symmetrical about this difference, ranging from +3 to -3 units (Fig 2). The between-visit differences in other CLIDE measures ranged from 0.02% to 4.60% (Table 2) and were also symmetrical. Only 2 subjects differed by 3 DEFC units between visits, with 117 subjects (90.00%) having a difference of either 0 (n = 84) or 1 (n = 33) unit. Scaling the other CLIDE measures to DEFC-equivalent units for comparison purposes, we found percentages of between-visit differences of ≤ 1 unit ranging from 55.38 to 80.77%. LoA for DEFC scores were [-1.62, 1.73], or [-32.40%, 34.60%]. Other CLIDE measures had lower LoA ranging from -35.50 to -58.40%, and upper LoA ranging from 37.00 to 55.80%. The CR for DEFC score was 0.42, or 8.47%, meaning that the true absolute difference between repeated DEFC scores under these conditions lies within less than half a unit (0.42) on the 5-point DEFC scale, with 95% probability. Overall, the DEFC showed good repeatability, comparable to or better than that of other CLIDE measures.

**Table 2:** Repeatability of the CLIDE diagnostic instruments.

	Limits of Agreement						Coefficient of Repeatability		n (%) Diffs w/in 1 DEFC Level Equiv.
	Raw Scale			% of Max					
INSTRUMENT	Mean Diff	Lower	Upper	Mean Diff	Lower	Upper	Raw Scale	% of Max	
**DEFC**	0.05	-1.62	1.73	1.00	-32.40	34.60	0.42	8.47	117 (90.00)
**NITBUT**	-0.46	-5.84	4.92	4.60	-58.40	49.20	0.14	1.38	81 (62.79)
**Distortion**	-0.01	-8.93	8.92	0.06	-55.80	55.80	8.72	54.50	72 (55.38)
**Wettability**	0.03	-1.42	1.48	0.75	-35.50	37.00	0.12	3.03	105 (80.77)
**Avg. Severity VAS**	-0.30	-50.56	49.96	0.30	-50.56	49.96	4.74	4.74	95 (73.08)
**Avg. Frequency VAS**	-1.08	-44.26	42.09	1.08	-44.26	42.09	30.45	30.45	92 (70.77)
**EOD Severity VAS**	1.24	-46.65	49.13	1.24	-46.65	49.13	0.70	0.70	94 (73.44)
**EOD Frequency VAS**	-0.02	-42.77	42.72	0.02	-42.77	42.72	0.31	0.31	98 (76.56)

The mean inter-visit (Visit 2 –Visit 1) difference in DEFC score (1.00%) was within the normal variation encountered with other CLIDE measures, which ranged from 0.02% to 4.60%. The coefficient of repeatability estimates that 95% of inter-visit differences will lie within less than half a DEFC level (0.42 on the 5-point scale). Ninety percent of inter-visit differences in DEFC score were within 1 level. The repeatability of the DEFC is comparable to that of the other CLIDE assessments.

*EOD = End Of Day*. *VAS = Visual Analog Scale*. *DEFC Level Equiv*. = *20% of the observed range of the variable (i*.*e*., *because 1 DEFC level is 20% of its own 5-point scale)*.

### DEFC score: Relationships to other CLIDE assessments

Subjects reported DEFC scores spanning the range of CLIDE symptoms (1–5), with a median score of 3, and a mean (SD) score of 2.8 (1.4), combined over the 2 visits. Table 3 shows the distribution of DEFC scores at each visit, along with the mean (SD) of each CLIDE assessment, stratified on DEFC score.

**Table 3:** DEFC score vs. other CLIDE assessments.

	DEFC SCORE				
	1	2	3	4	5
**n (%)**:	72 (27.7)	31 (11.9)	59 (22.7)	74 (28.5)	24 (9.2)
**Mean (SD)**:					
**Pre-Lens NITBUT**	3.97 (2.85)	3.15 (1.79)	2.73 (2.14)	2.38 (2.69)	1.86 (1.72)
**Pre-Lens Distortion**	9.60 (3.79)	9.87 (3.51)	9.17 (3.83)	9.74 (3.85)	9.83 (2.79)
**Pre-Lens Wettability**	2.95 (0.69)	2.77 (0.72)	2.92 (0.64)	2.82 (0.60)	2.97 (0.71)
**OSDI**	7.96 (8.55)	10.69 (14.06)	11.33 (10.30)	19.23 (12.78)	25.80 (17.24)
**Avg. Severity VAS**	18.91 (26.36)	21.67 (23.58)	35.89 (27.98)	50.55 (21.44)	48.00 (22.94)
**Avg. Frequency VAS**	13.91 (15.39)	25.54 (29.43)	40.37 (28.43)	56.10 (21.13)	60.12 (25.66)
**EOD Severity VAS**	25.32 (24.69)	37.16 (29.89)	52.32 (26.11)	70.66 (19.75)	68.37 (25.88)
**EOD Frequency VAS**	25.28 (24.68)	39.67 (33.65)	55.47 (28.11)	73.70 (19.29)	70.87 (28.55)

Shown are the sample sizes at each level of the DEFC (Visit 1 and Visit 2 combined; n = 260), along with the mean (SD) of each CLIDE assessment that was compared with the DEFC. Pre-lens NITBUT is shorter the higher the DEFC score, and all subjective ratings of symptoms are higher the higher the DEFC score.

*EOD = End-Of-Day*.

We found there to be significantly (p < 0.001) shorter pre-lens NITBUT (i.e., greater tear film instability) with higher DEFC score (i.e., worse symptoms). We examined a number of variables derived from pre-lens NITBUT on both the raw and natural log scales, and found that the best model fits were achieved using the worse pre-lens NITBUT of the two eyes, with three individual measurements above 10 sec truncated at that time to better approximate normality and avoid undue leverage effects. The pre-lens NITBUT ranged from approximately 4.0 sec on average among subjects with no CLIDE symptoms (DEFC score = 1) to 1.9 sec on average among subjects whose CLIDE symptoms are severe enough to frequently interfere with daily activities (DEFC score = 5). Table 4 shows the sensitivity and specificity of pre-lens NITBUT for our first DEFC-based diagnosis of CLIDE (No Symptoms vs. Any Symptoms) to be 65% and 50%, respectively, at a threshold of 2.5 sec. For our second DEFC-based diagnosis of CLIDE (No/Mild Symptoms vs. Debilitating Symptoms), the sensitivity and specificity of pre-lens NITBUT were 73% and 48%, respectively, at a threshold of 2.0 sec.

**Table 4:** Sensitivity and specificity of CLIDE assessments for two DEFC-based diagnoses.

**Diagnosis 1: No Symptoms vs. Any Symptoms**					
	**Sensitivity (%)**	**95% CI**	**Specificity (%)**	**95% CI**	**Threshold**
**Pre-Lens NITBUT**	65.4	[59.9, 70.9]	50.0	[40.4, 59.6]	≤ 2.5
**Pre-Lens Distortion**	50.3	[44.6, 56.1]	52.9	[43.3, 62.5]	≥ 12
**Pre-Lens Wettability**	48.6	[42.8, 54.4]	58.7	[49.2, 68.1]	≤ 2.5
**OSDI**	55.9	[50.2, 61.7]	80.8	[73.2, 88.3]	≥ 11.4
**Avg. Severity VAS**	65.7	[60.2, 71.2]	76.0	[67.7, 84.2]	≥ 21
**Avg. Frequency VAS**	72.3	[67.2, 77.6]	76.0	[67.7, 84.2]	≥ 21
**EOD Severity VAS**	71.3	[66.1, 76.6]	76.9	[68.8, 85.0]	≥ 45
**EOD Frequency VAS**	70.3	[65.0, 75.6]	81.7	[74.3, 89.2]	≥ 48
**Diagnosis 2: No/Mild Symptoms vs. Debilitating Symptoms**					
	**Sensitivity (%)**	**95% CI**	**Specificity (%)**	**95% CI**	**Threshold**
**Pre-Lens NITBUT**	73.2	[66.2, 80.2]	48.1	[41.7, 54.5]	≤ 2.0
**Pre-Lens Distortion**	52.3	[44.4, 60.2]	52.3	[46.0, 58.7]	≥ 12
**Pre-Lens Wettability**	50.3	[42.4, 58.2]	55.7	[49.4, 62.0]	≤ 2.5
**OSDI**	71.2	[64.1, 78.4]	70.0	[64.2, 75.9]	≥ 11.4
**Avg. Severity VAS**	82.4	[76.3, 88.4]	68.4	[62.4, 74.3]	≥ 24
**Avg. Frequency VAS**	74.5	[67.6, 81.4]	75.5	[70.1, 81.0]	≥ 42
**EOD Severity VAS**	73.2	[66.2, 80.2]	73.0	[67.3, 78.6]	≥ 63
**EOD Frequency VAS**	81.7	[75.6, 87.8]	67.5	[61.5, 73.5]	≥ 54

Sensitivities tended to be slightly higher for DEFC-based Diagnosis 2. Specificities for Diagnoses 1 and 2 tended to be about the same, or slightly lower for Diagnosis 2 (but not significantly so). With the exception of OSDI, thresholds were higher (i.e., symptoms ratings higher, NITBUT shorter) for identifying subjects with debilitating CLIDE symptoms.

*EOD = End-Of-Day*.

We did not find a significantly worse grade of distortion of the reflected topographer mires at 10 sec post-blink with higher DEFC score (Table 3). We examined a number of variables derived from grading of distortion, including the individual grades in each quadrant, and the sum distortion grade over all quadrants. We found no significant association between any distortion grade variable and DEFC score, with the sum score across quadrants from the worse eye (p = 0.540) having the best model fit, although all models were similar. With no significant association, grade of distortion was clearly not useful for distinguishing subjects with no symptoms from those with any CLIDE symptoms, having a sensitivity and a specificity of 0.50 and 0.52, respectively (Table 4); nor was grade of distortion useful for distinguishing subjects with no/mild symptoms from those with debilitating symptoms requiring intervention, with a sensitivity and a specificity of 0.52 and 0.52, respectively.

Clinician grading of in vivo contact lens wettability did not show a trend related to DEFC score (Table 3), and indeed in the mixed effects models was not a significant factor (e.g., p = 0.607 for the worse eye, that is, the eye with the less wettable lens). As expected, grade of lens wettability was not useful for distinguishing subjects with no symptoms from those with any CLIDE symptoms, having a sensitivity and a specificity of 0.48 and 0.58, respectively (Table 4); nor was grade of contact lens wettability useful for distinguishing subjects with no/mild symptoms from those with debilitating symptoms requiring intervention, with a sensitivity and a specificity of 0.50 and 0.55, respectively.

**Fig 3:**
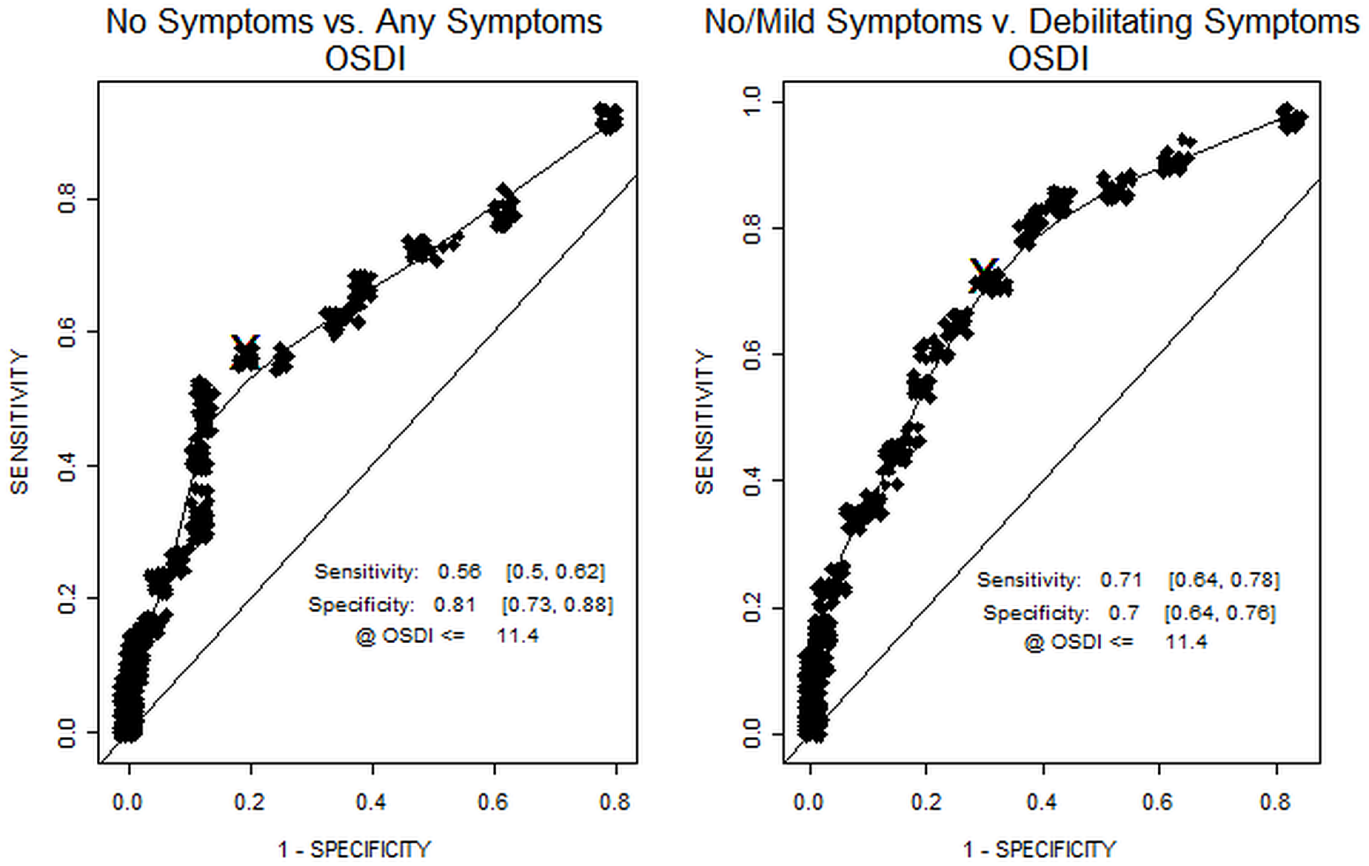
ROC curves for the OSDI. For the first DEFC-based CLIDE diagnosis, the OSDI showed a low sensitivity but a relatively high specificity. For the second DEFC-based diagnosis, the sensitivity of the OSDI improved with a small concomitant reduction in specificity.

The OSDI score was significantly related to the DEFC score (p < 0.001), and showed a consistent increase across the levels of the DEFC (Table 3) from a mean (SD) of 8.0 (8.6) among subjects with no CLIDE symptoms (DEFC score = 1) to 25.8 (17.2) among subjects with the most severe symptoms (DEFC score = 5). As shown in Fig 3, for the first DEFC-based CLIDE diagnosis (No Symptoms vs. Any Symptoms), the OSDI exhibited a low sensitivity of 0.55 but a relatively high specificity of 0.80 (Table 4). For the second DEFC-based diagnosis (No/Mild Symptoms vs. Debilitating Symptoms), the OSDI showed a sensitivity and specificity of 0.71 and 0.70, respectively.

**Fig 4:**
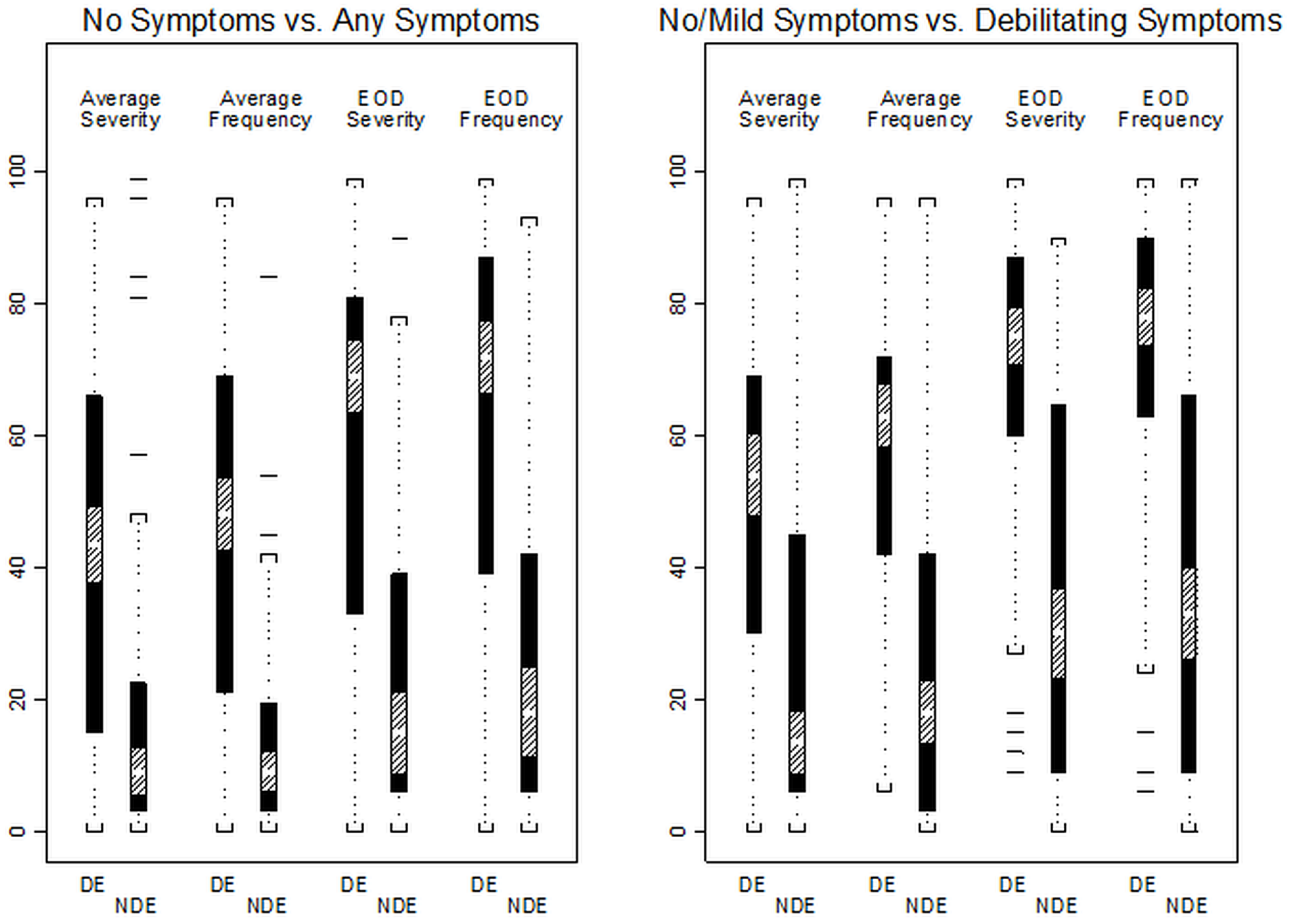
A trend of higher VAS ratings with higher DEFC score. EOD ratings tended to be slightly higher than on-average ratings. The subjective VAS ratings had higher sensitivities and specificities than did any of the clinical measurements, and were similar to those of the OSDI. *DE = Contact Lens-Induced Dry Eye (DEFC score ≥ 2); NDE = Non-DE (DEFC score = 1)*.

There were significant associations between DEFC score and subjective VAS ratings of symptoms, including on-average severity and frequency of dryness while wearing lenses, and end-of-day (EOD) severity and frequency of dryness (all p < 0.001). A clear trend of higher VAS ratings with higher DEFC score was found (Table 3), and EOD ratings tended to be slightly higher than on-average ratings (Fig 4). On-average dryness severity ranged from approximately 19 on the 100-point VAS scale for those with no symptoms (DEFC score = 1) to 48 for those with the most debilitating symptoms (DEFC score = 5), while EOD dryness severity ranged from approximately 25 to 68, on average, across those same DEFC categories. Similarly, on-average dryness frequency ranged in mean from approximately 14 to 60 across the DEFC score spectrum, while EOD dryness frequency means ranged from approximately 25 to 71. Sensitivities and specificities for our two DEFC-based diagnoses of CLIDE are shown in Table 4. In general, the subjective VAS ratings had higher sensitivities and specificities than did any of the clinical measurements, and were similar to those of the OSDI.

## Discussion

In this article, we have introduced the Berkeley Dry Eye Flow Chart, which has the potential advantages of being simple to understand, fast to administer to large numbers of subjects or patients, and provides a simple 1–5 score that directly reflects not only the presence of dryness symptoms and the level of discomfort they induce, but the functional impact of these symptoms on daily visual activities such as reading or using a computer. We have shown that the repeatability of the DEFC is comparable to or better than that of other common clinical and subjective measures of CLIDE. We have demonstrated a direct relationship between higher DEFC score and shorter pre-lens tear breakup time, higher VAS ratings of severity and frequency of symptoms, both on-average and at end-of-day, and higher OSDI score. Finally, we have demonstrated how the DEFC can be used to categorize subjects into CLIDE and non-CLIDE groups based on different diagnostic criteria, and estimated the sensitivity and specificity of each subjective and clinical CLIDE assessment to our two different DEFC-based diagnoses, along with the optimum diagnostic threshold for each assessment.

The DEFC was significantly related to, and showed clinically significant trends in the expected direction with, subjective ratings of symptoms using the VAS and the OSDI, and with pre-lens tear film instability measured by NITBUT. These are among the most commonly employed assessments for both CLIDE and DE in general. In contrast, grades of topographer mires distortion and in vivo contact lens wettability were not significantly related to DEFC score, and furthermore did not suggest a linear trend of worse distortion or poorer wettability with higher DEFC score. These assessments, however, are not standard tests for CLIDE or DE in clinical practice. We included them in this study because some previous work in our lab [39] and others’ [10,40] suggested that these might prove to be useful assessments related to severity of symptoms. This, however, turned out not to be the case in our study population. Thus the validation of the DEFC is based on the more commonly accepted assessments of tear film instability measurement and subjective ratings of symptoms.

The presence of a contact lens partitions the tear film into pre- and post-lens segments [41], and the pre-lens tear film is typically less stable than the pre-corneal tear film without lenses [42]. It remains equivocal whether pre-lens tear film stability differs between symptomatic and asymptomatic contact lens wearers. Estimates of average pre-lens NITBUT from asymptomatic hydrogel lens wearers range from 2.8 sec to 10.8 sec depending on lens type as well as on lens care solution formulation [43–46]. Pre-lens NITBUT has been found across a similar range in symptomatic subjects, and while symptomatic and asymptomatic subjects have been found to be significantly different in some studies [47], they have not in others [48,49]. Ours is the first study to show a distinct trend in pre-lens NITBUT *in vivo* across the range of CLIDE severity, from approximately 4 sec on average among subjects with no CLIDE symptoms to less than 2 sec among subjects with the most debilitating CLIDE. In addition, this study is the first to establish diagnostic thresholds (≤ 2.5 sec for any CLIDE symptoms, ≤ 2.0 sec for debilitating symptoms) for pre-lens, *in vivo* NITBUT (albeit at sensitivities and specificities of 65 and 50, respectively, for any CLIDE symptoms, and 73 and 48, respectively, for debilitating symptoms). It should be noted that the sensitivities and specificities, and the diagnostic thresholds obtained by ROC analysis in this study must be interpreted in the context of our study population: its demographic makeup, contact lens history, ocular characteristics, and so forth. For example, a much older population with substantially different refractive demands could very well elicit different diagnostic performance and different optimum diagnostic thresholds.

Corneal and conjunctival staining were also assessed at each exam. Although visible ocular surface damage can be indicative of severe, prolonged DE or CLIDE [50], in our study there were no significant relationships between DEFC score and any of our ocular surface staining variables. This is because, in our sample of young, healthy, experienced contact lens-wearing subjects, who wore fresh pairs of their habitual contact lenses for 6 hours only prior to assessment, there were very few cases of even moderate staining—certainly not sufficient to test any hypothetical statistical association.

The ROC analysis showed that even the best assessments for CLIDE do not operate with anything close to perfect sensitivity and specificity. In other words, even for the best assessments there will be false positives and false negatives. The sensitivities and specificities we found in this study are broadly in line with the relatively few examples published to date for CLIDE diagnosis, and with the somewhat more commonly reported diagnosis of DE in general. It is impossible to compare the sensitivities and specificities we found directly to other published examples because differences in study design, population characteristics, and in the criteria for determining a positive diagnosis all result in widely varying estimates across studies. For example, the sensitivities of NITBUT were 65% and 73% for our two DEFC-based diagnoses, respectively, compared with published examples ranging from 55% to 90%; the specificities for NITBUT that we found were 50% and 48%, while published examples ranged from 21% to 85% [51–55]. Studies employing various types of questionnaires have estimated sensitivities of 60% to 86%, compared with approximately 66% to 82% for our VAS questionnaire items, and specificities of 46% to 94%, compared with our range of approximately 68% to 82% [51,55–58]. A great many other potential diagnostic instruments (primarily for DE) have been investigated, including the Schirmer test, fluorescein tear breakup time, tear osmolarity, imprint cytology, vital staining, tear ferning, and a variety of subjective symptom instruments, leading to published estimates of sensitivity ranging from as low as 39% to as high as 98%, and estimates of specificity ranging from 10% to 100% [15,20,51,59–66]. The primary difference between studies is the set of criteria by which DE or CLIDE was defined, which renders direct comparisons of diagnostic performance uninformative. At the very least, the DEFC provides another means of defining CLIDE or DE, with certain practical advantages over other methods, and against which the most common diagnostic tools perform with sensitivities and specificities comparable to previous definitions of CLIDE or DE.

In the current study, sensitivities tended to be slightly higher for our second DEFC-based diagnoses (No/Mild Symptoms vs. Debilitating Symptoms), in which subjects with mild symptoms that do not cause discomfort sufficient to interfere with activities are not distinguished from subjects with no symptoms whatsoever. This shows that CLIDE assessments tend to be better at identifying subjects with debilitating symptoms than they are at distinguishing symptom-free subjects from those with any level of CLIDE symptoms from severe to mild. Specificities for both DEFC-based diagnoses of CLIDE tended to be about the same, or slightly lower for the second diagnosis (but not significantly so). With the exception of OSDI, thresholds were higher (i.e., symptoms ratings higher, NITBUT shorter) for subjects with debilitating CLIDE symptoms.

There were some differences particular to the OSDI in diagnosing CLIDE as based on our DEFC categorization. For the first DEFC-based diagnosis of CLIDE (No Symptoms vs. Any Symptoms), the OSDI had low sensitivity (56%), meaning that the OSDI score did not identify a number of subjects with DEFC ≥ 2 as being CLIDE-positive. On the other hand, it had high specificity (81%), meaning that it is better at correctly identifying subjects with DEFC = 1. OSDI scores above the ROC threshold of 11.4 are probably characteristic mostly of those with CLIDE symptoms severe enough to cause significant discomfort [15] and possibly affect visual functioning in daily activities. Thus a number of subjects with only mild symptoms not affecting daily activities had OSDI scores that, while somewhat elevated over the truly CLIDE-negative subjects, did not exceed the ROC threshold and therefore were not diagnosed as CLIDE-positive. This is supported by the higher sensitivity (71%) of the OSDI for the second DEFC-based diagnosis of CLIDE (No/Mild Symptoms vs. Debilitating Symptoms), while still maintaining a specificity (70%) that is not significantly lower than for the first diagnosis. It is interesting to note that the ROC analysis-generated threshold for diagnosing CLIDE using the OSDI in our study population (≥ 11.4) is very close to the commonly accepted clinical threshold OSDI score (≥ 13) for diagnosing DE [67,68].

Our results illuminate a common difficulty in identifying optimal diagnostic criteria for multifaceted conditions such as CLIDE, arising from a common misconception. There are a number of published reports covering various clinical, laboratory and subjective assessments shown to be related to a CLIDE outcome. Often these variables are shown to be significant explanatory variables in multivariable association models of CLIDE, whether the outcome be presence/absence, risk, or severity of symptoms. However, a significant association with CLIDE does not necessarily make such an assessment a good diagnostic indicator [38]. It would seem natural to assume, from perusing the literature, that if certain questionnaire items or clinical assessments are consistently shown to be significantly related to CLIDE outcomes, they should therefore be routinely administered to ascertain a patient’s CLIDE status; however, without directly examining the diagnostic performance of such assessments (i.e., sensitivity and specificity; determination of threshold values), there is no way of knowing whether or to what extent such assessments are useful as diagnostic indicators for CLIDE.

**Fig 5:**
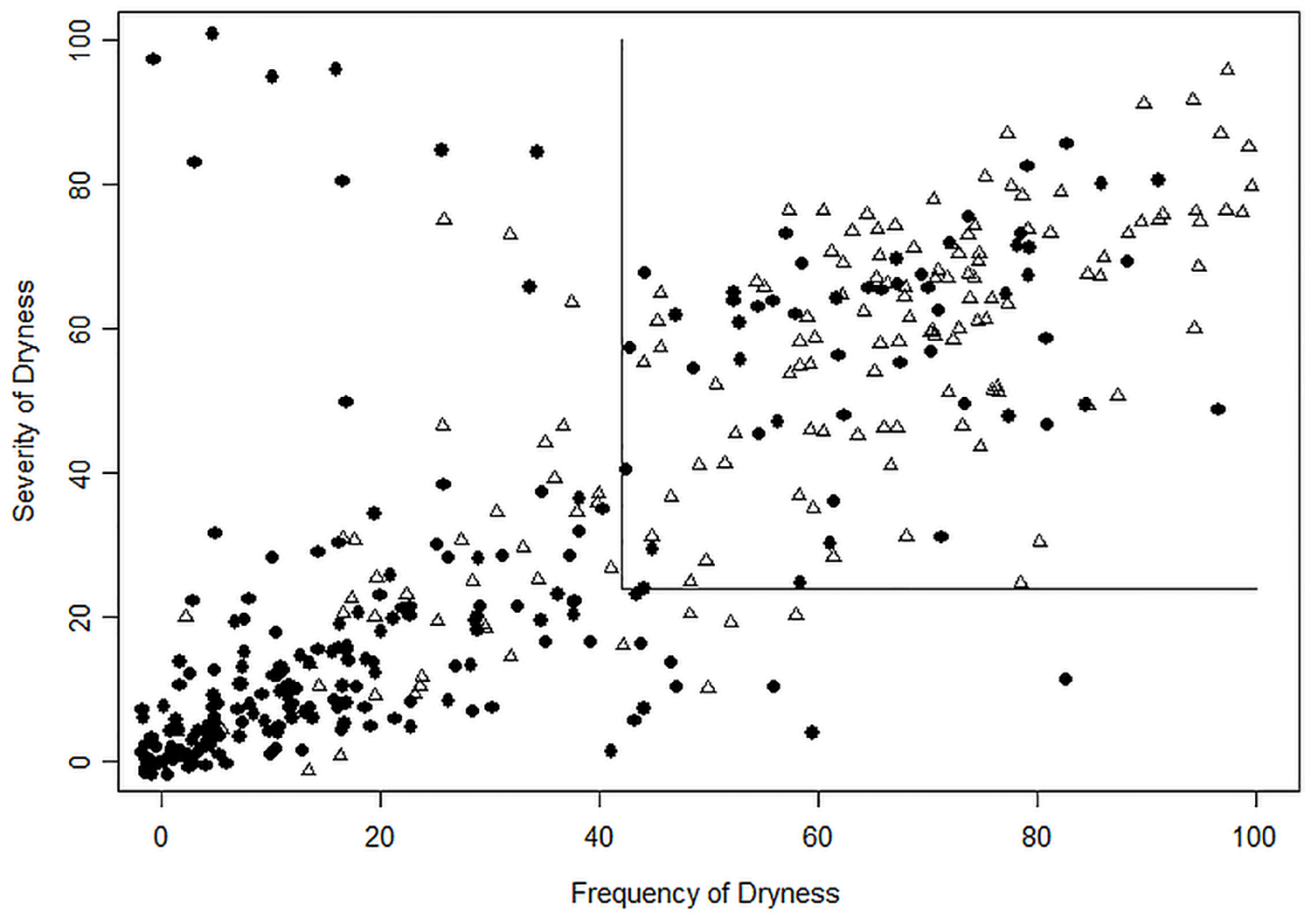
Potential pitfalls in using assessments that are significantly associated with CLIDE outcomes as diagnostic instruments. There is clear separation between CLIDE-negative (filled circles) and CLIDE-positive (open triangles) subjects; however, there are subjects with VAS ratings above the diagnostic thresholds (No/Mild Symptoms vs. Debilitating Symptoms thresholds shown) who do not have CLIDE (false-positives), as well as those with VAS ratings below the thresholds who do have CLIDE (false-negatives).

Fig 5 depicts the potential pitfalls in using assessments that are significantly associated with CLIDE outcomes as diagnostic tools: while there is clear separation between CLIDE-negative (filled circles in the figure) and CLIDE-positive (open triangles) subjects, there are nevertheless a number of subjects with symptom severity and/or frequency above the optimum thresholds (as determined by ROC analysis) who did not self-classify as having CLIDE, as well as those with symptom ratings below the thresholds who do self-classify as CLIDE sufferers. These “false positives” and “false negatives” contribute to the diagnostic performance—the sensitivities and specificities—of the VAS rating scale symptom assessments.

It is important to understand that the ordinal categorical nature of the DEFC gives the instrument inherently good repeatability but relatively poor discrimination ability, or resolution. In other words, it would require a relatively large change in a patient’s or subject’s symptoms before there was a change in DEFC score, compared with, say, the 100-point VAS rating scale which has relatively poor repeatability but can record differences or changes in symptoms with much finer resolution. The uses for which the DEFC is designed make this appropriate: a clinician or researcher would not use the DEFC to determine whether there were fine between-subject differences in symptoms (e.g., that would show up on the VAS rating scale), but rather, would use the DEFC to determine whether differences in symptoms are sufficient to represent qualitatively different levels in terms of a patient’s or subject’s quality of life (e.g., dryness symptoms that do or do not cause significant ocular discomfort; symptoms that do or do not interfere with visual activities in daily life). While both types of instruments have a place in the clinical and research arenas, these distinctions highlight the need to employ the subjective response instrument that is most appropriate to the specific clinical or research application.

In this study, we compared individual assessments of CLIDE used in current research and clinical practice to our new metric, the Berkeley DEFC score. In practice, most clinicians would consider a number of different assessments before identifying a subject as having CLIDE or some other DE-related condition [50]. It is possible that the best diagnosis of CLIDE consists of a suite of clinical and subjective measures, each with its own diagnostic threshold, that collectively would provide the highest sensitivity and specificity for a test of CLIDE. Although there has been some limited work done in this area [51,52], the collective performance of commonly employed suites of diagnostic tools, the identification of the best set of assessments for diagnosis of CLIDE or DE in different populations, and determination of the optimum diagnostic thresholds for those assessments have not been well-studied to date. Work is ongoing in this area.

## Conclusions

This article has introduced the Berkeley Dry Eye Flow Chart, discussed its potential advantages as a CLIDE screening tool, demonstrated its association with several known CLIDE assessments, and established its repeatability. In addition, we have demonstrated that the 5-level ordinal categorization of CLIDE based on the DEFC corresponds directly to trends in VAS ratings of symptoms, OSDI scores, and pre-lens NITBUT, and we have established the optimum diagnostic thresholds for these commonly employed assessments in our population by ROC analysis. We believe that the DEFC can provide a fast, accurate, functional categorization of CLIDE that takes into account the extent to which symptoms affect visual activities in daily life, and that it could prove a useful addition to the set of tools available to researchers and clinicians working with symptomatic contact lens wearers.
